# Plant cell wall lignification and monolignol metabolism

**DOI:** 10.3389/fpls.2013.00220

**Published:** 2013-07-09

**Authors:** Yin Wang, Maxime Chantreau, Richard Sibout, Simon Hawkins

**Affiliations:** ^1^Unite Mixte de Recherche 1318, Institut Jean-Pierre Bourgin, Institut National de la Recherche Agronomique, Saclay Plant SciencesVersailles, France; ^2^Lille 1 UMR 1281, UniversitéLille Nord de FranceVilleneuve d’Ascq, France; ^3^Unite Mixte de Recherche 1281, Stress Abiotiques et Différenciation des Végétaux Cultivés, Institut National de la Recherche AgronomiqueVilleneuve d’Ascq, France

**Keywords:** monolignol, lignin, lignan, metabolism, biomass

## Abstract

Plants are built of various specialized cell types that differ in their cell wall composition and structure. The cell walls of certain tissues (xylem, sclerenchyma) are characterized by the presence of the heterogeneous lignin polymer that plays an essential role in their physiology. This phenolic polymer is composed of different monomeric units – the monolignols – that are linked together by several covalent bonds. Numerous studies have shown that monolignol biosynthesis and polymerization to form lignin are tightly controlled in different cell types and tissues. However, our understanding of the genetic control of monolignol transport and polymerization remains incomplete, despite some recent promising results. This situation is made more complex since we know that monolignols or related compounds are sometimes produced in non-lignified tissues. In this review, we focus on some key steps of monolignol metabolism including polymerization, transport, and compartmentation. As well as being of fundamental interest, the quantity of lignin and its nature are also known to have a negative effect on the industrial processing of plant lignocellulose biomass. A more complete view of monolignol metabolism and the relationship that exists between lignin and other monolignol-derived compounds thereby appears essential if we wish to improve biomass quality.

## INTRODUCTION

The majority of plant biomass consists of different cell wall polymers produced by living plant cells. In most cases, these polymers are energy-rich linked sugars that form the major structural components in plant cell walls, particularly in the thick secondary cell walls that characterize certain tissues. In addition to polysaccharides, another major cell wall polymer – lignin – limits access to cell wall sugars and negatively affects human utilization of biomass (livestock feed, paper manufacturing, and lignocellulosic biofuel production; Chapple et al., 2007). Because of its significant economic impact and central role in higher plant development, lignification is an important topic in plant biochemistry.

Despite a few exceptions (Martone et al., 2009), lignin is a phenolic biopolymer that is only detected in vascular plants. It is generated by radical coupling of hydroxycinnamyl alcohols named monolignols: coniferyl alcohol, sinapyl alcohol, and *p*-coumaryl alcohol. When introduced in the lignin polymer the corresponding monolignols are named guaiacyl (G), sinapyl (S), and hydroxyl-coumaroyl (H) units, respectively. The biosynthesis of monolignols is initiated from the general phenylpropanoid pathway (Dixon et al., 2001). Although tyrosine was proposed to be the starting point of phenylpropanoid metabolism in some plants such as grasses (Neish, 1961; Higuchi, 1990), it is generally recognized that monolignols are derived from phenylalanine via a series of enzymatic reactions, catalyzed by the following enzymes: phenylalanine ammonia lyase (PAL), cinnamate 4-hydroxylase (C4H), 4-coumarate coenzyme A ligase (4CL), ferulate 5-hydroxylase (F5H), *p*-coumarate 3-hydroxylase (C3H), *p*-hydroxycinnamoyl-CoA:quinate/shikimate hydroxycinnamoyl transferase (HCT), caffeoyl-CoA *O*-methyltransferase (CCoAOMT), cinnamoyl-CoA reductase (CCR), caffeic acid *O*-methyltransferase (COMT), and cinnamyl alcohol dehydrogenase (CAD; Vanholme et al., 2009).

The composition of lignin varies between plant species and tissues. In general, lignins from gymnosperms and related species are rich in G units and contains low amounts of H units, whereas dicot lignins are mainly composed of G and S units (Weng and Chapple, 2010). It is important to note that differences in monolignol content are controlled by key enzymes that are often limiting (e.g., F5H is limiting for S unit production; Weng and Chapple, 2010). Consequently, certain natural genotypes/varieties may show unusual proportions of monolignols as illustrated by the recent discovery of a *Populus nigra* variety containing a truncated HCT enzyme and producing substantial amounts of H units (Vanholme et al., 2013) that are usually almost completely absent in poplar lignin. Similarly, a loblolly pine naturally affected in cinnamyl alcohol dehydrogenase activity and showing modified lignin structure was also detected 20 years ago in a natural population (MacKay et al., 1995; Ralph et al., 1997).

**FIGURE 1:**
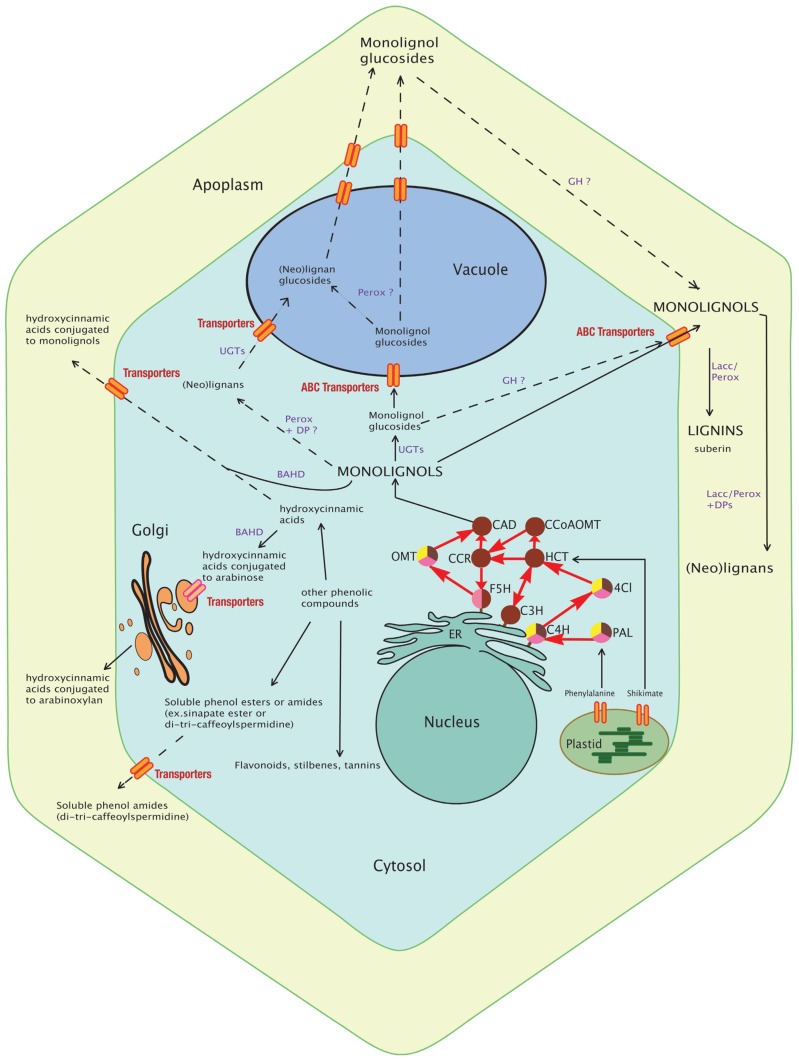
**Schematic view of the monolignol biosynthetic pathway.** The synthesis of monolignols from phenylalanine and shikimate involves cytosolic (PAL, HCT, 4CI, CCR, CAD, CCoAOMT, and OMT) and ER membrane-anchored (the cytochrome P450 enzymes F5H, C3H, and C4H) enzymes. Monolignols and lignans may be conjugated by UGTs and then transported to the vacuole or directly transported to the cell wall for oxidative cross-linking by apoplastic peroxidases and laccases into lignins. In grasses, some hydroxycinnamic acids are conjugated to arabinose by BAHD enzyme for export via the Golgi apparatus and future incorporation into polysaccharides (pectins or arabinoxylans) whereas some are conjugated to monolignols (mainly sinapyl alcohol) in the cytosol and incorporated into lignin. Other phenolic compounds (soluble phenolic esters and amides, flavonoids) are shown and may indirectly interact with lignification. Their production implies common steps (enzymes) with the monolignol pathway. Most of these transport routes and storage forms still remain to be discovered and the localization of some enzymes (GH, Perox, Lacc) confirmed. The dashed lines delineate putative pathways and full lines delineate known routes. Enzymes of the monolignol pathway are represented by circles. The brown color indicates involvement of the enzyme in the monolignol pathway; yellow indicates involvement in the flavonoid pathway; and pink indicates involvement in the sinapate ester pathway. PAL, phenylalanine ammonia lyase; C4H, cinnamic acid 4-hydroxylase; 4CL, 4-hydroxycinnamoyl-CoA ligase; HCT, hydroxycinnamoyl-CoA:shikimate hydroxycinnamoyl transferase; C3H, *p*-coumaroyl shikimate 3′-hydroxylase; CCoAOMT, caffeoyl-CoA *O*-methyltransferase; CCR, hydroxycinnamoyl-CoA reductase; F5H, ferulic acid 5-hydroxylase; COMT, caffeic acid/5-hydroxyferulic acid *O*-methyltransferase; CAD, cinnamyl alcohol dehydrogenase; UGT, UDP-glycosyltransferase; GH, beta-glucosidase; Perox, peroxidase; Lacc, laccase; DPs, dirigent proteins; BAHD, hydroxycinnamic acid transferase; ER, endoplasmic reticulum.

From a developmental point of view, lignification is generally initiated during the formation of the secondary cell wall although cell-/tissue-specific differences in the developmental pattern can occur. For example, lignification is initiated in the compound primary cell wall of xylem elements (middle lamella/cell corners) at the start of secondary cell wall formation (Donaldson, 2001; Fromm et al., 2003). In contrast, lignin may be deposited at later stages in other tissues (Boerjan et al., 2003; Baghdady et al., 2006; Harrington et al., 2012). For instance, parenchyma cells present in the secondary xylem of *Arabidopsis* hypocotyls are devoid of lignin during the vegetative stage (Sibout et al., 2008). This period may range over months according to different *Arabidopsis* accessions but ends as soon as the plant start flowering when parenchyma cells start to reinforce their secondary cell walls by producing lignin. Parenchyma therefore develops into sclerenchyma, a tissue dedicated to mechanical support. Biotic- and/or abiotic-stresses can also induce lignification in the walls of cells that do not normally lignify under non-stress conditions (Srivastava et al., 2007; Kim et al., 2008). Compression wood in coniferous trees also contains significant amounts of *p*-coumaryl alcohol (Sjöström, 1993). The observation that lignin biosynthetic genes are up-regulated (or activated) following mechanical injury suggests that stress lignification is a tightly controlled phenomenon. In contrast, the expression of monolignol biosynthesis genes is not always correlated with the presence of the lignin polymer. Although this is quite surprising at first glance, this situation can be explained by the fact that a wide range of other (non-lignin) products (hydroxycinnamic acids, phenols esters and phenol amides, or lignans; **Figure 1**) are also produced by the phenylpropanoid pathway.

In summary, lignification is a dynamic, flexible process reinforcing cell walls according to the different needs (water conduction, mechanical support, defense) of the plant during its life. Nevertheless, once produced, lignins (and the associated carbon) remain anchored in the cell wall since plants do not possess the enzymatic machinery necessary to recycle this polymer in contrast to other cell wall polysaccharides such as (1,3;1,4)-beta-D-glucans for instance (Fincher, 2009). The irreversibility of this situation also underlines the necessity for tight regulation of the lignification process.

Metabolic engineering can be used not only to reduce or modify natural lignin structure, but can also be employed to produce novel lignin structures in biomass crops with the goal of improving its structure for biomaterial or bioethanol production (Dixon et al., 1996; Vanholme et al., 2012a). Nevertheless, as underlined by a recent systems biology approach that investigated the consequences of lignin perturbations in *Arabidopsis* mutants, it is clear that our knowledge of this complex process is far from complete and that modifications in the lignification process can be accompanied by unexpected changes in gene expression and metabolism (Vanholme et al., 2012b). In this review, we focus on another aspect of lignin biology where our understanding is only partial – the different processes that occur after monolignol biosynthesis, including the acylation and glycosylation of monolignols in the cytoplasm, their transport through the membrane to the apoplast, and their deglycosylation, and polymerization into lignins. We also discuss lignans and related compounds produced by the same biosynthetic pathway. In this review we aim to point out some gaps in our knowledge of lignification that are potentially limiting for biomass use and engineering.

## TRANSPORT AND COMPARTMENTATION

Monolignols are synthesized in the cytoplasm and translocated to the cell wall for subsequent polymerization (Alejandro et al., 2012). For many years, the mechanism of transport remained unknown and different hypotheses were suggested (passive diffusion, exocytosis, active transport, etc.; Liu et al., 2011; Liu, 2012). An elegant biochemical study has recently demonstrated that the glycosylation status determines monolignol transport and subcellular compartmentation (Miao and Liu, 2010). Plasma membrane-derived vesicles prepared from *Arabidopsis* and poplar cells transported coniferyl alcohol (the aglycone form), but not coniferin (the glycosylated form of coniferyl alcohol), in an ATP-dependant manner, whereas tonoplast-derived vesicles transported the glycosylated form but not the aglycone. Taken together these results would suggest that coniferyl alcohol is transported into the cell wall across the plasma membrane in an ATP-dependant process by an ABC-transporter and is subsequently polymerized by laccases (LACs) and/or peroxidases (PRXs). In contrast, the glycosylated form (e.g., coniferin) is transported into the vacuole for storage. Genetic confirmation of the involvement of ABC transporters in monolignol transport into the cell wall was recently reported (Alejandro et al., 2012). Co-expression studies in *Arabidopsis* identified an ABCG transporter gene (*AtABCG29*) co-regulated with phenylpropanoid gene expression and that was expressed in lignin-containing organs and tissues. Protein-fusion studies demonstrated that AtABCG29 was localized in the plasma membrane and transport studies in yeast suggested that the protein was involved in transporting *p*-coumaryl alcohol, but not coniferyl alcohol. A minor activity was also observed toward sinapyl alcohol. *Abcg29* mutant lines showed reduced root growth when grown on medium containing *p*-coumaroyl alcohol, but not coniferyl alcohol or sinapyl alcohol supporting the idea that AtABCG29 is involved in p-coumaryl alcohol transport. Lignin analyses of roots from two mutant lines revealed decreases in the amounts of H, G, and S subunits as compared to WT roots, an unexpected result since AtABCG29 is highly specific for *p*-coumaryl alcohol and it would be expected that only H units would be reduced. Interestingly, the mutant lines also showed metabolomic modifications in soluble phenolics, flavonoids and glucosinolates together with reductions in associated gene expression. These modifications are reminiscent of changes observed in different lignin mutants (Vanholme et al., 2012b) underlining the fact that phenylpropanoid metabolism can be perturbed not only at the biosynthesis level, but also by modifications of monolignol transport. The results also raise the possibility that the observed cell-specific variation in lignin composition (e.g., vessel element walls are rich in G lignin whereas fiber walls contain S–G lignin) may also be regulated via specific ABC transporters. Plant genomes contain large ABC transporter gene families (e.g., 130 genes in *Arabidopsis*) but ABC transporters for coniferyl alcohol and sinapyl alcohol remain to be identified and characterized. In addition, it is probable that monolignol glucosides from the vacuole participate to tracheary element lignification following cell death and membrane disruption (Pesquet et al., 2013). Additional information on the potential role(s) of ABC transporters in the lignification process is discussed by Sibout and Hofte (2012).

## MONOLIGNOL GLYCOSYLATION AND DEGLYCOSYLATION

Glycosylation of monolignols is catalyzed by UDP-glycosyltransferases (UGTs) belonging to the glycosyltransferase (GT) family 1. These enzymes play an important role in stabilization, enhancement of water solubility and deactivation/detoxification of a wide range of natural products including hormones and secondary metabolites (Lim and Bowles, 2004). A recent phylogenetic reconstruction (Caputi et al., 2012) of family 1 UGTs reveals a dramatic evolutionary increase in the number of family 1 GTs from one in the model green alga *Chlamydomonas* to 243 in poplar reflecting the increased complexity of plant life on Earth. Less than 20% plant UGTs have been functionally characterized (Yonekura-Sakakibara et al., 2012). A major problem is that plant GTs may be redundant. Functional analyses in *Arabidopsis* (Lanot et al., 2006) of the UGT72E clade suggested that the *UGT72E2* gene product was responsible for monolignol glycosylation. Chemical analyses of soluble phenolics in light-grown roots from the triple mutant showed a significant decrease in the quantity of coniferyl alcohol- and sinapyl alcohol-glucosides as compared to WT plants. Over-expression of the *UGT72E2* gene resulted in a 10-fold increase in coniferin levels and a lower increase in sinapyl alcohol glucoside in roots suggesting that this is the principal gene involved in monolignol glycosylation in *Arabidopsis*. Interestingly, rosette leaves accumulated sinapoyl glucoside to higher levels than coniferyl alcohol glucoside hinting at organ-specific variations in glycosylation mechanisms. However, no impact on lignin content/composition was observed. Evidence suggesting a link between lignification and monolignol glycosylation was recently demonstrated (Wang et al., 2012) when the over-expression in tobacco plants of a poplar UGT (*PtGT1*) homolog to the *Arabidopsis* *UGT72E1-3* genes was shown to be associated with an increased lignification as well as early flowering. Nevertheless, the observation that recombinant PtGT1 showed no activity toward monolignols and the fact that transformed plants did not contain higher amounts of monolignol glycosides led the authors to suggest that the observed impact of *PtGT1* over-expression in tobacco was indirect. Further research is clearly needed to identify not only the UGTs specifically involved in monolignol glycosylation, but also to demonstrate a functional link between modifications in *UGT* gene expression and lignification. The potential importance of monolignol glycobiology in regulating lignin biosynthesis is also complicated by the fact that, in addition to glucose, plant UGTs can also use different sugars (glucose, rhamnose, galactose, glucuronic acid, etc.) to glycosylate monolignols (Caputi et al., 2012).

If monolignol glucosides give rise to monolignols that are then incorporated into the lignin polymer, then enzymes (beta-glucosidases) should be co-localized with coniferin and syringin. Beta-glucosidases capable of hydrolyzing monolignol glucosides were first identified in cell wall fractions of *Picea abies* hypocotyls and roots (Marcinowski and Grisebach, 1978). Subsequently, a beta-glucosidase showing high affinity for coniferin was co-localized with PRX activity to cell walls of young differentiating xylem in *Pinus contorta* (Dharmawardhana et al., 1995,^1999^). In Angiosperms, beta-glucosidase activity was first located in the cell walls of *Cicer arietinum* L. (Hosel et al., 1978; Burmeister and Hosel, 1981). The co-localization of beta-glucosidase activity and monolignol glucosides in differentiating xylem of conifers and some angiosperms led to the hypothesis that monolignol glucosides could represent lignin precursors. However, subsequent work in *Ginkgo biloba* (Tsuji et al., 2005) showing that a beta-glucosidase demonstrated a 10 times greater affinity toward coniferaldehyde as compared to coniferin threw doubt on this initial idea. Subsequent work with radioactive precursors indicated that coniferyl alcohol derived from coniferin was only weakly incorporated into the lignin polymer and that greater amounts were converted into coniferaldehyde glucoside followed by subsequent deglycosylation and CAD-mediated conversion of the coniferaldehyde into coniferyl alcohol. These results suggested that coniferin represented the storage form of coniferyl alcohol and was not the direct precursor of coniferyl alcohol for lignin. In *Arabidopsis*, three orthologs (BGLU45/At1g61810; BGLU46/At1g61820; BGLU47/At4g21760) of the *Pinus contorta* beta-glucosidase gene are present and belong to the group 10 of the *Arabidopsis* GH1 family (Xu et al., 2004). Heterologous expressions of BGLU45 and BGLU46 showed that BGLU45 is highly specific for the three monolignol glucosides, whereas BGLU46 preferentially uses *p*-coumaryl alcohol glucoside as well as showing broad activity toward other phenolic glucosides (Xu et al., 2004; Escamilla-Trevino et al., 2006). A recent functional study of *Arabidopsis* *bglu45-1* and *bglu46-2* T-DNA mutants (Chapelle et al., 2012) showed only slight reductions (8.5 and 6.5%, respectively) in global beta-glucosidase activity and protein amounts as estimated by Western blots following Concanavalin-A column chromatography, presumably because of functional redundancy – at least four other glucosidases are expressed in *Arabidopsis* stems – and antibody cross-reactivity. Lignin analyses showed a slight but significant increase in Klason lignin for two BGLU45 mutants of the WS accession (*bglu45-1* and *bglu45-3*) under long-day conditions only but not in Col0 bglu45 mutants. No significant modification in lignin content was observed for *bglu46* mutants. Similarly, thioacidolysis revealed no changes in lignin subunit structure. Metabolomics (ultra performance liquid chromatography–mass spectrometry, UPLC –MS) identified around 100 different small glycosylated phenolic compounds, most of which were increased in the mutants. For the 10 most important differentials, five were identified/partially identified and all were derived from G units. Interestingly, an important ecotype effect was observed with, for example, an approximate 20-fold increase of coniferin in WS *bglu45-1* mutants, but only a 3.4-fold increase in Col10 mutants. While coniferyl alcohol, sinapyl alcohol, coniferaldehyde, sinapaldehyde, and syringin levels were not significantly modified, certain (neo)lignans increased [lariciresinol hexoside or isodihydrodehydrodiconiferyl alcohol (IDDDC) hexoside, dehydrodiconiferyl alcohol (DDC) hexoside]. Based on these observations, the authors proposed that monolignol glucosides are the storage form of monolignols and not the direct precursors. Nevertheless, it is interesting to note that *BGLU45* and *BGLU46* expression are deregulated under various biotic stresses suggesting that the monolignol storage pool might be used under pathogen attack in the fabrication of “defense lignin” and/or phytoalexins. Certainly, such a hypothesis allows a better understanding of the basic “spatial problem” associated with beta-glucosidase enzyme and substrate compartmentation (**Figure 1**). As indicated above, the *Pinus contorta* beta-glucosidase was localized to differentiating xylem walls and BGLU45/46 proteins are secreted to the cell wall (Chapelle et al., 2012), whereas glycosylated monolignols are stored in the vacuole. Under such conditions it is perhaps not surprising that beta-glucosidase gene down-regulation has little/no effect on lignin levels under standard conditions and that the critical step occurs at the glycosylation step deciding whether monolignols are targeted to the cell wall without glycosylation for lignification, or glycosylated for storage in the vacuole. In contrast, cell and vacuole disruption following pathogen attack, insect/herbivore feeding and potentially severe abiotic stress (cell freezing, desiccation) or during programed cell death (xylem vessel) would lead to contact between the monolignol glucosides and beta-glucosidases at the cell wall where PRXs and/or LACs could rapidly polymerize the aglycone forms. The role of beta-glucosidases in producing a wide range of bioactive defense molecules has been largely documented in plants (Morant et al., 2008).

Taken together, these observations indicate that the glycosylation/deglycosylation of monolignols play key roles in determining their availability for subsequent lignin biosynthesis thereby representing potentially interesting engineering targets for plant biomass improvement. In addition, similar mechanisms are also likely to affect the availability of ferulic- and *p*-coumarylic acids for lignin–hemicellulose/pectin and polysaccharide–polysaccharide covalent cross-links in the cell wall underlining the necessity for further research into the glycosylation/deglycosylation of monolignols and phenolic molecules.

## DIMERIZATION OF MONOLIGNOLS AND SYNTHESIS OF (NEO)LIGNANS

Monolignols can also give rise to more than 3,000 different lignans and associated structures such as neo-, sesqui-, and flavanolignans (Schmidt et al., 2010). Lignans are optically active phenylpropanoid dimers that result from the stereo-selective dirigent protein-mediated coupling of the 8 and 8′ C-atoms of two hydroxycinnamyl alcohol (monolignol) moieties (Davin et al., 1997). (Note: two different nomenclatures are used to describe the propane carbon atoms involved in monolignol linkages in the lignin polymer and in oligolignols. In oligolignols, the numbers 7, 8, and 9 are used whereas in the lignin polymer α, β, and γ are used to describe the same carbon atoms. A β–β lignin linkage therefore corresponds to an 8–8 *oligolignol* linkage). Lignan formation has been widely studied since these molecules are natural anti-oxidants, possess anti-microbial activity and have numerous beneficial effects on human (Ford et al., 2001). Lignans are known to play a role in the durability, longevity, and resistance of the heartwood of many tree species against wood-rotting fungi and are believed to function as phytoalexins in other (non-woody) plant species (Pohjamo et al., 2003). The formation and accumulation of lignans in flax (*Linum usitatissimum* L.) seeds has been particularly studied since they are one of the richest natural grain sources of certain lignans. The genus *Linum* contains approximately 200 species worldwide that can be divided into two main groups depending upon the major lignan types accumulated – either cyclolignans belonging to the aryltetralin series (AT lignans) or cyclolignans of the aryldihydronaphthalene (ADN)/arylnaphthalene (AN) group (Schmidt et al., 2010). Some plants in both groups can also accumulate dibenzylbutyrolactone (DBBL) lignans such as matairesinol and furofuran (FF) lignans such as pinoresinol. Both FF and DBBL lignans are precursors of the AT/ADN/AN lignans. In the seeds of flax, dimerization of two E-coniferyl alcohol molecules leads to the formation of 8S,8′S-(-)-pinoresinol, which is sequentially converted into 8S,8′S-(-)-lariciresinol and 8S,8′S-(++)-secoisolariciresinol via the action of the enzyme pinoresinol lariciresinol reductase (PLR). (+)-Secoisolariciresinol is then glycosylated to secoisolariciresinol diglucoside (SDG) that accumulates to high levels in flax seeds (linseed; Ford et al., 2001). The complexity of lignan stereochemistry was underlined in a recent study (Hemmati et al., 2010) that isolated two PLRs (PLR-Lu1, PLR-Lu2) showing opposite enantiospecificity. PLR-Lu1 utilizes 8S,8′S-pinoresinol, whereas PLR-Lu2 uses 8R,8′R-pinoresinol. Interestingly, gene expression analyses have shown that the Lu*PLR1* gene is expressed in different flax seed tissues, whereas the Lu*PLR2* gene is expressed in flax stem and leaf tissues (Hano et al., 2006; Hemmati et al., 2010; Venglat et al., 2011). In agreement with this organ-specific gene expression, 8S,8′S-SDG accumulates in flax seeds whereas aerial green parts accumulate 8R,8′R-SDG. Further analyses (Schmidt et al., 2012) of lignans in 16 different *Linum* species suggested that lignan stereochemistry also depended upon the species analyzed and whereas R,R-SDG was mainly accumulated in the seeds from species containing relatively high amounts of ADN/AN cyclolignans, S,S-SDG was accumulated in seeds from species containing only trace amounts of cyclolignans.

**FIGURE 2:**
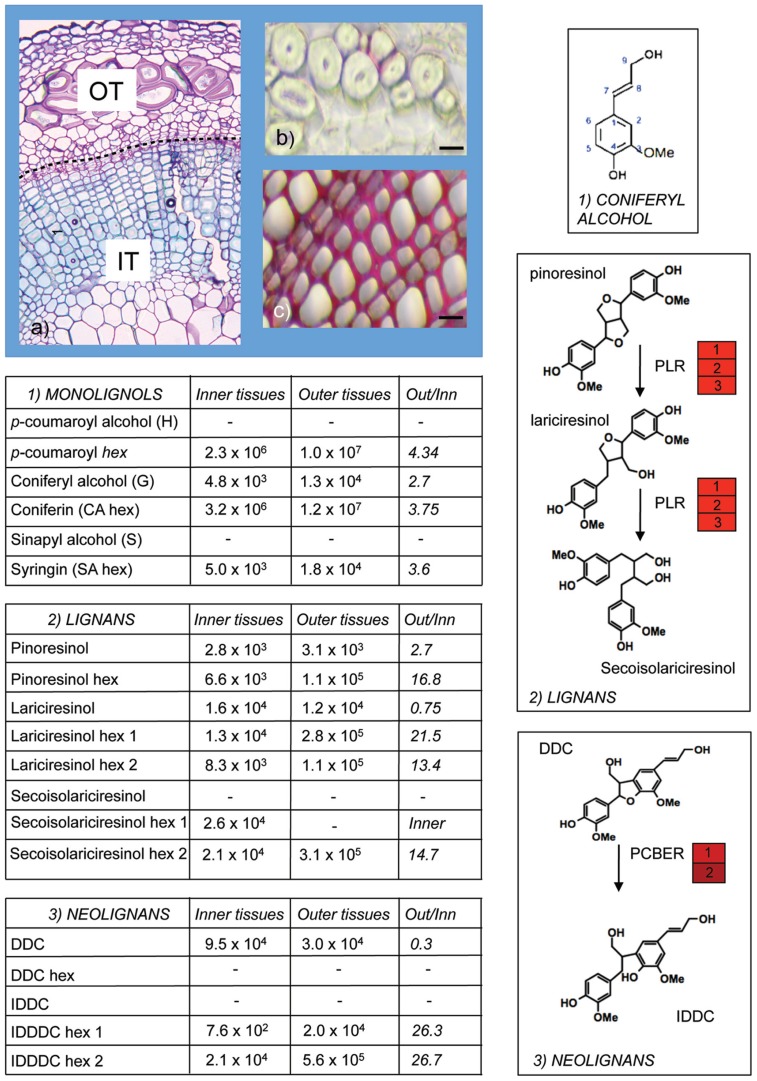
**Monolignol metabolism in flax stems.** Olignolignols were extracted from inner (IT) and outer (OT) tissues of flax stems **(A)** and analyzed by ultra-high performance liquid chromatography–Fourier transform–ion cyclotron resonance–mass spectrometry (UHPLC–FT–ICR–MS; Huis et al., 2012). Outer tissues are characterized by the presence of bast fibers with thick cellulose-rich secondary cell walls and extremely low lignin levels as indicated by limited red coloration with phloroglucinol-HCl staining **(B)**. Inner tissues contain xylem tissue with heavily lignified cell walls **(C)**. Aglycone and glycosylated forms of monolignols (1), lignans (2), and neolignans (3) were detected in both inner- and outer-stem samples. Glycosylated forms were generally more abundant in outer stems. DDC, dehydrodiconiferyl alcohol; IDDDC, isodihydrodehydrodiconiferyl alcohol; PLR, pinoresinol lariciresinol reductase; PCBER, phenylcoumaran benzylic ether reductase. Red heat blocks indicate corresponding unigene expression in inner- versus outer-stem tissues. This Figure illustrates the extremely rich metabolism post-monolignol biosynthesis. **(A)** Courtesy of Anne-Sophie Blervacq, Université Lille 1.

A further insight into monolignol/lignan biology in flax was provided by a combined transcriptomics and metabolomics study (Huis et al., 2012) of flax inner- (xylem rich) and outer- (bast fiber rich) stem tissues (**Figure 2**). More than eighty different lignans and neolignans including pinoresinol, lariciresinol, secoisolariciresinol, DDC, and IDDDC, as well as their glycosylated forms were detected in both tissues. In agreement with the presence of lignans and neolignans in the flax stem, transcripts corresponding to PLR, as well as the enzyme phenylcoumaran benzylic ether reductase (PCBER) catalyzing the reduction of DDC into IDDDC and previously identified as the most abundant protein in poplar xylem (Gang et al., 1999; Vander Mijnsbrugge et al., 2000) were also highly abundant. Although *PCBER* transcripts were more highly abundant in inner stem tissues, IDDDC was not detected in either inner- or outer-stem tissues, despite the presence of DDC in both tissues. In contrast IDDDC hexosides were detected in both stem tissues suggesting that the neolignan is rapidly glycosylated. Overall, the glycosylated forms were more abundant in outer stem tissues in comparison with inner stem tissues. Further work in other species is necessary to know whether such differences in (neo)lignan glycobiology are widespread and also the potential biological role. Interestingly, recent reports showing that lignans are detected in the phloem tissues (Ramos and Kato, 2012) and honey-dew of the stink-bug and that glycosylated flax lignans but not the aglycone forms significantly increased aphid *Myzus persicae* insect mortality (Saguez et al., 2012) raise the possibility that monolignol-derived compounds might be glycosylated and transported in phloem cells as a defense mechanism against insect predators.

The abundance of glycosylated (neo)lignans in flax and other species suggests the existence of different UGTs capable of glycosylating lignans. Lignan UGT activity was first identified in sesame (Noguchi et al., 2008) and more recently in *Forsythia* (Ono et al., 2010). Subsequent screening of a *Forsythia* cDNA library and biochemical characterization of recombinant UGT71A17 and UGT71A18 proteins showed that both proteins were capable of glycosylating (+)-pinoresinol albeit UGT71A17 activity was much weaker than UGT71A18 activity. Further characterization of the latter showed that it was active against different FF and DBBL lignans. Expression analyses indicated that both genes were expressed in leaves, floral buds, and petals, but that only *UGT71A18* was expressed in cell suspension cultures. It would obviously be interesting to see whether *UGT* genes are also expressed in other organs/tissues. Monolignol dimerization is presumably catalyzed by class III PRXs that are localized in the cell wall and/or vacuole suggesting that lignans should also be localized in these compartments. Although one study (Attoumbre et al., 2010) localized lignans to the cell wall in flax seeds, a positive signal was also observed in cytoplasmic inclusions raising the possibility that lignans might also be present in this compartment. Plant UGTs, unlike their mammalian counterparts, do not possess peptide signals and are therefore probably localized in the cytoplasm. In this case, where does (neo)lignan glycosylation take place – do plants possess plasma membrane/tonoplast ABC transporters capable of transporting neolignans into/out of cells/tonoplasts? In conclusion, although we now have a relatively good understanding of monolignol biosynthesis (even if our knowledge of transcriptional and post-transcriptional regulation processes is far from complete), it is clear that we have much to learn about the mechanisms regulating the availability of synthesized monolignols in the cell wall for subsequent lignification. A better understanding of these mechanisms should lead to the elaboration of new targets for engineering more efficient plant biomass.

## POLYMERIZATION

**FIGURE 3:**
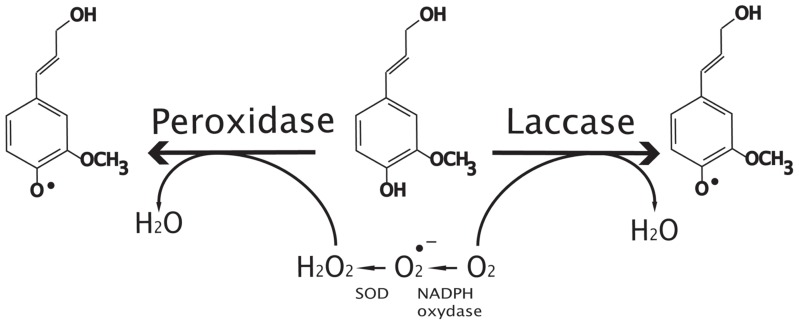
**Oxidative radicalization of monolignols by peroxidases and laccases.** Peroxidases use peroxide produced by super oxide dismutase proteins (SOD) and NADPH oxidase as co-substrate to make oxidative radicalization of phenols (coniferyl alcohol in this case) whereas laccases use oxygen. Note that peroxidase can oxidize both monolignols and oligomers to produce oxidized phenols.

**FIGURE 4:**
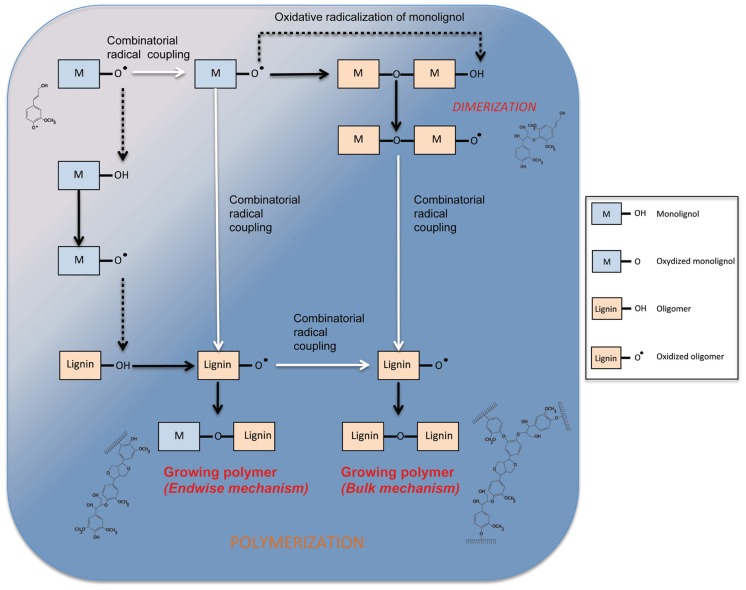
**Mechanism of phenol oxidation and polymerization in the cell wall.** Dotted lines show transfert of radicals between phenols (monolignol or oligomer). White arrows represent combinatorial radical coupling between phenols. Dark arrows show the product of radical–radical coupling and the oxidation of a phenol.

Beyond dimerization formation of lignins requires a polymerization step. The complex structure of the polymers built up of different subunits (H, G, and S), the presence of different types of linkages between subunits and the absence of repeated motifs suggest a highly unpredictable polymerization mechanism probably affected by its microenvironment (pH, polysaccharides, etc.; Wingender et al., 1999). In the developing cell wall, lignin polymerization occurs by radical coupling reactions (Freudenberg, 1959; Figures 3 and 4). Type III PRXs and LACs are probably both involved in monolignol oxidation and participate to lignification (**Figure 3**). PRXs involved in lignification are exported to the apoplasm in lignifying tissues where monolignol oxidation takes place (Koutaniemi et al., 2005; Vanholme et al., 2010). The oxidation reaction uses H_2_O_2_ as co-substrate which is probably provided by combined action of NADPH oxidase or germin-like proteins, even though firm genetic evidence for the involvement of these enzymes is currently lacking (Davidson et al., 2009). PRX exist as large multigene families (73 and 138 in *A. thaliana* and *Oryza sativa*, respectively; Welinder et al., 2002) making the clarification of the biological role of each member a considerable challenge. Clear characterization is also complicated by the fact that PRXs generally show low substrate specificities (Hiraga et al., 2001). Nevertheless recent studies suggesting that some PRX isoforms may be more specialized in the polymerization of guaiacyl or/and syringyl units could help to explain how plants are able to control lignin monomeric structure (Barcelo et al., 2007; Martinez-Cortes et al., 2012). Both the isoelectric point of the proteins and specific motifs in the PRX primary structure could support protein–substrate interaction with one monomer type over another and therefore influence lignin composition. Two examples of genetic manipulation illustrate this hypothesis. Antisense suppression of one PRX gene led to a global reduction of both G and S units in tobacco (Blee et al., 2003) whereas analyses of PRX down-regulated poplar (Li et al., 2003) revealed that lignin contained less G units but that S content was unchanged. More recently, Herrero et al. (2013a) used *Zinnia elegans* PRX protein sequence data to identify *Arabidopsis* orthologs for functional characterization. Histochemical analyses of the *atprx72* mutant suggest that it has reduced overall lignin levels and a low syringyl unit content as compared to WT plants (Herrero et al., 2013b). However, the mutant shows a strong developmental phenotype and it would be necessary to confirm this result on a second mutant allele. In parallel, Shigeto et al. (2013) used similar strategies to functionally characterize other PRX gene candidates in *Arabidopsis*. Chemical analyses indicated a significant decrease in the total lignin content and modified lignin structure in *atprx2* and *atprx25* mutants and altered lignin structure in *atprx71* mutants.

The mechanism of oxidative radicalization of phenols followed by combinatorial radical coupling is well described (Vanholme et al., 2010; van Parijs et al., 2010). Nevertheless, the kinetics of monomer incorporation into the lignin polymer by PRX remains poorly understood. In a recent article, Demont-Caulet et al. (2010) propose a general scheme for S–G copolymerization coordinated by a PRX (PRX34) purified from *A. thaliana*. The purified enzyme shows a high substrate specificity and seems unable to directly oxidize S monomers in the absence of G units. The authors studied kinetics of the monomer conversion and subsequent formation of di-, tri-, and tetramers by using monomer feeding experiments. Their results suggest that endwise and bulk polymerization mechanisms are likely to co-exist within the same system, the bulk mechanisms occurring after total consumption of the monomers. During endwise polymerization, monomers are added to the polymer one-by-one whereas during bulk polymerization different oligomers are linked together at the same time (**Figure 4**). Interestingly, Demont-Caulet et al. (2010) show that while monomer availability may influence the relative preponderance of the two mechanisms, it is the nature of the monomer that exerts the most influence. Indeed, under *in vitro* oxidation conditions, the presence of S monomer converted the PRX34-mediated polymerization of G monomers from a bulk to an endwise mechanism in agreement with data from Argyropoulos et al. (2002). These observations underline the central role of PRXs in controlling lignin biosynthesis. In a recent article, the group of Niko Geldner proposed a very nice model where PRXs are the last pieces of a puzzle that orchestrate lignification in Casparian strips in *Arabidopsis* roots (Lee et al., 2013). The authors show that the PRX enzymes colocalize with the very specific Casparian strip domain protein (CASP) and that their activity is indirectly under the control of an NADPH oxidase named respiratory burst oxidase homolog F protein (RBOHF) that provides H_2_O_2_ to PRXs via the action of an unknown superoxide dismutase (SOD). These fascinating results show for the first time that a *trans*-membrane protein (CASP) is able to control peroxidase activities thereby representing a potential mechanism for localized spatial control of lignification.

Therefore, PRXs represent interesting targets for modifying not only lignin content but also lignin structure, even if high redundancy due to the existence of large gene families complicates genetic approaches. It is also important to emphasize that PRXs exported to the extracellular matrix of some tissues may not only be involved in lignification. For instance, they may participate to suberin polymerization, cell membrane protection or else, catalyze di-ferulate bonds between polysaccharides particularly in grasses where arabinoxylans are highly feruloylated (Fry, 2004; Arrieta-Baez and Stark, 2006). Tannins that contribute to the heartwood color in some trees (e.g., walnut), as well as lignans (see above) may also be produced by PRXs.

Laccases are a large group of copper-containing glycoproteins in plants, bacteria, and fungi. In contrast to PRXs, LACs use O_2_ instead of H_2_O_2_ to oxidize the monolignols (**Figure 3**). Although there is not enough evidence to confirm that plant LACs are localized in the cell wall, the presence of signal peptides in the N-terminal protein region suggests a subcellular localization (Cesarino et al., 2013) whereas some PRXs could be detected in other compartments. A number of *in vitro* experiments have identified different putative LAC substrates including flavonoids, 4-Hydroxyindole, coniferyl alcohol, *p*-coumaryl alcohol, and sinapyl alcohol (Mayer and Staples, 2002). While certain studies confirm that tree LACs can polymerize monolignols *in vitro* (Sterjiades et al., 1992; Ranocha et al., 2002), the broad substrate specificity of plant LAC needs to be confirmed *in vivo*. LACs, like PRXs, belong to (smaller) multigenic families in plants and different members may have redundant or specific biological functions. This is based upon two contrasting observations showing that in single antisense poplar plants with reduced LAC transcripts, neither lignin content nor composition is significantly altered (Ranocha et al., 2002). In contrast, the transgenic poplars exhibited an increase in total soluble phenolic content and perturbation of xylem fiber integrity (Ranocha et al., 2002). In *Arabidopsis*, 17 LACs have been reported (McCaig et al., 2005) but until 2011, only indirect evidence linked these enzymes to lignification in *Arabidopsis*. *AtLAC2* mutants showed a reduced root elongation under PEG treatment suggesting a possible impact on vasculature (Cai et al., 2006) and *AtLAC4* mutants under continuous light conditions were associated with a weak irregular xylem phenotype (Brown et al., 2005). Similarly, the *Arabidopsis* *AtLAC15* mutant was identified during a transparent testa screen since it possesses seeds with a pale brown seed coat due to altered flavonoid condensation (Pourcel et al., 2005). Mutants also showed a 59% increase of soluble proanthocyanidins when compared to wild-type and the extractable lignin content in *tt10* seeds was reduced by nearly 30% (Egertsdotter et al., 2004). This observation would suggest that both monolignols and proanthocyanidins could serve as substrates for AtLAC15 in *Arabidopsis* seed coats. Transcriptomics (Ehlting et al., 2005) indicate that four LACs (*AtLAC4*, *AtLAC11*, *AtLAC12*, *AtLAC17*) show moderate or high expression levels in *Arabidopsis* developing stems. These four genes are up-regulated during the transition from young to intermediate stage stems and were therefore considered as good targets for modifying lignin.

Direct evidence showing that specific LACs are involved in monolignol polymerization *in planta* was obtained by the characterization of *Arabidopsis* *lac4lac17* double mutants (Berthet et al., 2011). The stems of double mutants possess collapsed vessels and show reduced G lignin deposition in fibers. Mutants also show a 20–40% decrease in total lignin content and a 130% increase in saccharification yields when compared to the wild-type control. Lignin composition (higher S/G ratio) is also modified and is mainly due to the decrease of G lignin in fibers. This is not surprising since LAC and lignin genes are tightly co-expressed (Berthet et al., 2011) and promoter-GUS analyses showed that *LAC4* is expressed in vascular bundles and interfascicular fibers of stems while *LAC17* is specifically expressed in fibers (Berthet et al., 2011). Phylogenetic analyses of *Arabidopsis* LACs showed that *LAC4* and *LAC17* are the best homologs (McCaig et al., 2005) suggesting redundant or at least similar activity for both proteins in lignification. Recently, Cesarino et al. (2013) identified a sugarcane LAC gene, *SofLAC*, putatively involved in lignification. Its transcripts are localized in the inner- and outer-lignified zones of stem sclerenchymatic bundle sheaths. *SofLAC* was expressed preferentially in young internodes and the corresponding transcript level decreased along with stem maturity. The *Arabidopsis* *lac17* mutant was partly complemented by expressing *SofLAC* under the control of the AtLAC17 promoter. *SofLAC* expression restored the lignin content but not the lignin composition in complemented *lac17* mutant lines. These results suggest that specific LACs may also play a role in lignification in monocots.

Studies on mutants affected in different specific steps of monolignol biosynthesis indicate that plants are able to incorporate substantial amounts of components (e.g., aldehydes, 5-hydroxyconiferyl alcohols) that are poorly incorporated in “normal” conditions. Currently, it is not known whether incorporation is mediated by PRXs or LACs, or by both proteins. Some attempts have also been made to engineer plants so as to incorporate unusual compounds (e.g., rosmarinic acid or hydroxybenzaldehyde and hydroxybenzoate derivatives) into their lignins (Eudes et al., 2012; Tobimatsu et al., 2012). The incorporation of such specific compounds has been proposed as a strategy to reduce the degree of lignin polymerization. The incorporation of novel compounds together with the existence of large LAC and PRX gene families showing broad substrate specificities raises exciting new possibilities for the production of modified lignins and improved biomass.

## ACYLATION

Plant lignins often contain various acids (acetates, *p*-hydroxybenzoate, and *p*-coumarate) in addition to conventional monolignols. It is usually accepted that some of them (ferulate, *p*-coumarate) are by-products or intermediates of the monolignol pathway since there is no evidence that an independent pathway exists. Several observations suggest that the degree of acylation could be reasonably expected to impact on biomass quality. First, amounts of acylated monomers show high natural variations between different species. Kenaf and Agave possess up to 60 and 80% of acylated lignins, respectively when compared to maize and wheat that show 18 and 3% of acylated lignins, respectively (reviewed by Withers et al., 2012). Second, it is possible that acylation may affect lignin structure during polymerization as well as subsequent degradation by chemical or biochemical processes such as saccharification and digestibility. In grasses, *p*-coumarates are linked to syringyl units and plants with low coumarate levels also generally show modifications in their S, or S and G content (Harrington et al., 2012). Importantly, coumaroylation of cell wall polysaccharides is most likely (or at least partly) catalyzed in the cytosol prior to export and polymerization in the cell wall strongly suggesting the existence of specific monolignol transferases and transporters (Withers et al., 2012; Molinari et al., 2013; **Figure 1**).

Until recently, the genetics underlying coumarylation of both lignin and arabinoxylans remained unknown, mainly because of the lack of suitable plants (specifically engineered to modify acylation) for functional characterization. It is generally accepted that the genes associated with such reactions would encode BAHD acyl-CoA transferase proteins. Recent work showing that transgenics have higher or lower feruloylation levels has suggested that some members of the BAHD acyl-CoA transferase protein family may be responsible for catalyzing the addition of ester-linked coumarate to cell wall arabinoxylans (Fernando et al., 2009; Withers et al., 2012; Bartley et al., 2013; Molinari et al., 2013). A member of the BAHD acyl transferase family was recently identified in the rice genome (Withers et al., 2012). Analyses of the recombinant protein produced in *E. coli* showed that it had a specific coumaroyl transferase activity. This enzyme OsPMT (*O. sativa* *p*-coumaric acid monolignol transferase) catalyzes the acylation of a monolignol with *p*-coumaric acid via *p*-coumaroyl CoA and the corresponding transcript is co-expressed with the monolignol biosynthetic *4CL* gene. Based on catalytic efficiency and reaction rate, the authors show that OsPMT would produce more *p*-coumaryl *p*-coumarates in saturating conditions but would favor the synthesis of sinapyl *p*-coumarate over coniferyl *p*-coumarate *in planta*. Interestingly this gene is grass specific and its enzymatic hallmark therefore perfectly correlates with the high level of coumaroylated sinapyl alcohols in these types of plant. In addition, the BAHD protein accepts coumarate, but not ferulate as a substrate. The production of rice plants with disrupted *OsPMT* expression may be difficult because of gene redundancy since there are at least two paralogs in this species. Nevertheless, it is interesting to note that there are less paralogs in *Brachypodium* (Withers et al., 2012) and the recent article published (Molinari et al., 2013) could help to identify suitable candidate genes for further study. The authors established a correlation between candidate *p*-coumaric acid monolignol transferase transcript levels and amounts of bound ferulate in different organs at different developmental stages. They propose different candidate genes similar to OsPMT genes to be responsible for the lignin bound *p*-coumaric acid.

## CO-EXPRESSION DATA MAY HELP TO SCREEN FOR PROTAGONIST INVOLVED IN THE LAST STEPS OF LIGNIN BIOSYNTHESIS

**FIGURE 5:**
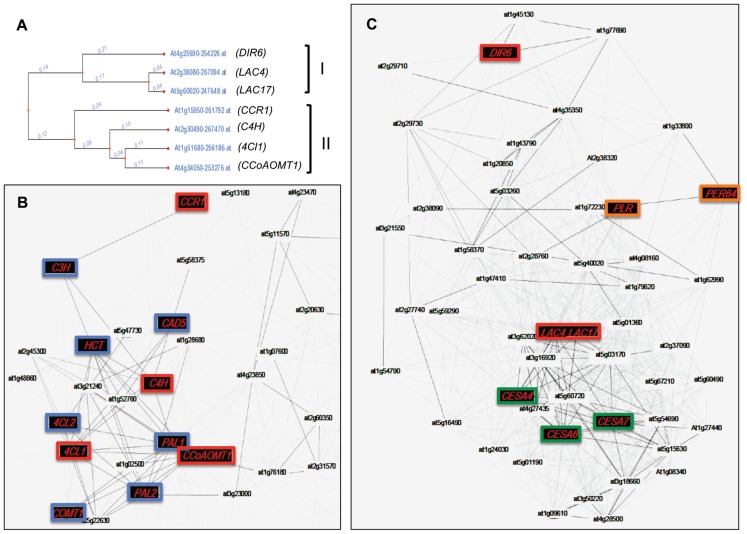
**Co-expression network of genes involved in lignin and lignan biosynthesis in *Arabidopsis*.** **(A)** Dendrogram representing the co-expression of genes involved in the biosynthesis and polymerization of monolignols. Two separate clusters (II and I, respectively) are obtained using the tool “expression tree” from Genecat (http://genecat.mpg.de). **(B)** Co-expressed gene network of genes present in cluster II. **(C)** Co-expressed gene network of genes present in cluster I. Nodes and edges represent genes and significant positive co-expression relationships between genes. Red nodes represent genes used as baits to produce gene co-expression networks. Blue nodes depict genes known to be involved in monolignol biosynthesis. Green nodes represent specific genes involved uniquely in cellulose synthesis of secondary cell walls. Orange nodes represent genes putatively involved in polymerization and lignan formation. DIR6, dirigent protein 6; LAC4, laccase 4; LAC17, laccase 17; CBR1, hydroxycinnamoyl-CoA reductase 1; C4H, cinnamic acid 4-hydroxylase; 4Cl, 4-hydroxycinnamoyl-CoA ligase 1; CCoAOMT1, caffeoyl-CoA *O*-methyltransferase; PLR, pinoresinol–lariciresinol reductase; PER64, peroxidase 64; CESA4, 7, 8, cellulose synthase 4, 7, and 8. Other abbreviations are given in **Figure 1**.

The last 20 years have seen the elucidation of the complete monolignol biosynthetic pathway and a number of limiting enzymes have been identified. Importantly, the expression of monolignol biosynthesis genes is not always correlated with the presence of the lignin polymer. This observation can be attributed to the fact that the monomers can also be used to generate a wide range of monolignol-derived compounds located in intra- or extracellular compartments and underlines the complexity of plant monolignol metabolism and regulation (**Figure 1**). One illustration of this phenomenon can be seen with the use of co-expression tools. Recent years have seen the emergence of large transcriptomic data sets and co-expression tools have been developed by different research groups (Ruprecht and Persson, 2012). These tools allow the identification of genes putatively involved in the same biological process as, in general, the expression of related genes should be transcriptionally coordinated (Usadel et al., 2009). Two recent articles have used this approach to analyze lignin biosynthesis in *Arabidopsis* and *Brachypodium* (Ruprecht and Persson, 2012). When monolignol biosynthesis genes (*4CL*, *C4H*, *CCoAOMT*) and monolignol polymerization/isomerization genes (*Dirigent protein 6*, *LAC4*, *LAC17*) are used as baits for the co-expression analysis with genecat (http://genecat.mpg.de/), two distinct gene networks clearly appear (**Figure 5**). LAC genes are clearly co-expressed with the cellulose synthase genes *CESA4*, *7*, and *8*, that are specific to secondary-, but not primary-cell wall formation. This co-expression is even stronger than that observed with *4CL*, *C4H*, and *CCoAOMT* that are all highly co-expressed with other monolignol biosynthetic genes such as *PAL*, *COMT*, *CAD*, *CCR*, *C3H*, and *HCT* (**Figure 5**). Since the monolignol biosynthetic pathway shares steps with the general phenylpropanoid pathway involved in the production of various non-lignin compounds (Goujon et al., 2003; Bassard et al., 2010), the co-expression data could suggest that *LAC4* and *LAC17* might represent a more efficient bait than monolignol biosynthesis genes when trying to identify other “lignin-related” genes including those encoding transporters and other polymerization enzymes.

## CONCLUSION

It is clear that the metabolism and polymerization of both monolignols and other more unusual monomers following their biosynthesis are more complex than previously thought. A number of key potential regulation points are involved in these processes allowing plants to rapidly respond to different developmental and environmental cues and that could also constitute interesting novel engineering targets for optimizing lignin production and composition. This will be achieved by answering a long list of crucial questions. For instance, where are glucosidases located – in the cell wall, in the vacuole or in the cytosol? In which conditions (development, stress, etc.) are they active? How are glycosylated monolignols released from the vacuole? What are their roles? Where does the dimerization occur for lignan formation? Are cytosolic PRXs involved? How do lignin polymers grow in the cell wall ? What are the specific roles of LACs versus PRXs? Are CASP-like proteins also present in other lignified tissues (xylem and sclerenchyma for instance)? The regulation of lignification has long been studied and modified by scientists at the monolignol biosynthetic level only, often with the sole goal of reducing lignin contents. It is now becoming clear that many other important control points exist that will allow the development of novel strategies for improving plant biomass for both energy and material production. We can reasonably expect that some of these key questions will be answered during the next decade.

## Conflict of Interest Statement

The authors declare that the research was conducted in the absence of any commercial or financial relationships that could be construed as a potential conflict of interest.
